# Elucidation of the unique mutation spectrum of severe hearing loss in a Vietnamese pediatric population

**DOI:** 10.1038/s41598-018-38245-4

**Published:** 2019-02-07

**Authors:** Jae Joon Han, Pham Dinh Nguyen, Doo-Yi Oh, Jin Hee Han, Ah-Reum Kim, Min Young Kim, Hye-Rim Park, Lam Huyen Tran, Nguyen Huu Dung, Ja-Won Koo, Jun Ho Lee, Seung Ha Oh, Hoang Anh Vu, Byung Yoon Choi

**Affiliations:** 1 Department of Otorhinolaryngology-Head and Neck Surgery, Seoul National University Bundang Hospital, Seongnam, Korea; 2 Department of Otorhinolaryngology, Children’s Hospital 1, Ho Chi Minh City, Vietnam; 3 Biomedical Research Institute, Seoul National University Hospital, Seoul, Korea; 4 Department of Otorhinolaryngology, University of Medicine and Pharmacy at Ho Chi Minh City, Ho Chi Minh City, Vietnam; 5 Department of Otorhinolaryngology-Head and Neck Surgery, Seoul National University Hospital, Seoul, Korea; 6 Center for Molecular Biomedicine, University of Medicine and Pharmacy at Ho Chi Minh City, Ho Chi Minh City, Vietnam

## Abstract

The mutational spectrum of deafness in Indochina Peninsula, including Vietnam, remains mostly undetermined. This significantly hampers the progress toward establishing an effective genetic screening method and early customized rehabilitation modalities for hearing loss. In this study, we evaluated the genetic profile of severe-to-profound hearing loss in a Vietnamese pediatric population using a hierarchical genetic analysis protocol that screened 11 known deafness-causing variants, followed by massively parallel sequencing targeting 129 deafness-associated genes. Eighty-seven children with isolated severe-to-profound non-syndromic hearing loss without family history were included. The overall molecular diagnostic yield was estimated to be 31.7%. The mutational spectrum for severe-to-profound non-syndromic hearing loss in our Vietnamese population was unique: The most prevalent variants resided in the *MYO15A* gene (7.2%), followed by *GJB2* (6.9%), *MYO7A* (5.5%), *SLC26A4* (4.6%), *TMC1* (1.8%), *ESPN* (1.8%), *POU3F4* (1.8%), *MYH14* (1.8%), *EYA1* (1.8%), and *MR-RNR1* (1.1%). The unique spectrum of causative genes in the Vietnamese deaf population was similar to that in the southern Chinese deaf population. It is our hope that the mutation spectrum provided here could aid in establishing an efficient protocol for genetic analysis of severe-to-profound hearing loss and a customized screening kit for the Vietnamese population.

## Introduction

The prevalence of infants born with moderate to severe bilateral hearing impairment is one in 1000, and the prevalence of infants with profound hearing loss, which causes severe speech impairment requiring cochlear implantation, is 4 in 10,000^1^. It has been shown that genetic causes account for about half of all known causes of these congenital hearing loss cases^2,3^. Hence, understanding the genetic etiology of hearing loss could aid practitioners to get a better understanding of the progression and risk of hearing loss in children, ultimately leading to the development of an optimal intervention strategy^4–6^. Most children with hearing loss were born to parents with normal hearing^7^, and genetic counseling for auditory rehabilitation and future family planning might be needed.

However, molecular genetic diagnosis of congenital hearing loss is not always feasible. Deafness is an extremely heterogeneous disorder; to date, more than 400 various types of syndromic hearing loss as well as more than 163 chromosomal loci and 115 genes of non-syndromic hearing loss (NSHL) have been identified (http://hereditaryhearingloss.org as of Apr 2018). It has been reported that not only are there numerous genetic variants responsible for hearing loss, but also the prevalence of the genetic variants is variable according to ethnicity and country of the study population. *GJB2* variants are the most common cause of NSHL, and its detection rate differs depending on the ethnicity and country of origin; 20% in Caucasians of European descent^8^, 43% in Israelis^9^, 21~27% in Japan and Chinese^10–12^, 17% in Tunisians^13^, 17% in Iranian^14^, 14% in Australians^15^, and 10% in Koreans^3,16^. Furthermore, the *GJB2* mutations were rarely found in African patients (0~1.0%)^17–19^. As such, the prevalence of *GJB2* variants in hearing loss varied widely across different regions even within a single country. According to several genetic studies performed in Iran^20^, the prevalence of *GJB2* varied from 0% to 38.3%, depending on the region. This diversity reflects the ethnic footprint of the Iranian population from neighboring countries. *SLC26A4* (4.8~18%), *MYO15A* (9.6%), and *MYO7A* (~5%) were other prevalent causative genes in the Iranian population with NSHL^20^. In East Asian countries, mutations of *SLC26A4* (10~13%) and mitochondrial DNA (1.8~2.1%) were frequently found following the mutation of *GJB2*^12,21^. The mutations of *GJB2*, *USH2A*, *MYO15A*, and *STRC* genes were frequently found in the European population with autosomal recessive hearing loss^22^.

Knowing the mutational spectrum of deafness in a certain population offers a background data for the establishment of molecular genetic screening protocol for that population. The mutational spectrum of hearing loss in the population of Indochina Peninsula has not been studied yet; to the best of our knowledge, only information regarding the prevalence of *GJB2* variants in Thailand is available^23,24^. Vietnam is the easternmost country on the Indochina Peninsula, sharing the region with Thailand, Laos, and Cambodia. In this study, we utilized the hierarchical genetic screening protocol that proved to be effective in East Asian populations to evaluate the genetic profile of severe-to-profound NSHL in the Vietnamese population, which in turn could provide the basis of health policy and medical processes for the treatment of hearing loss in the region.

## Results

### Three step protocol for genetic analysis

**Figure 1:**
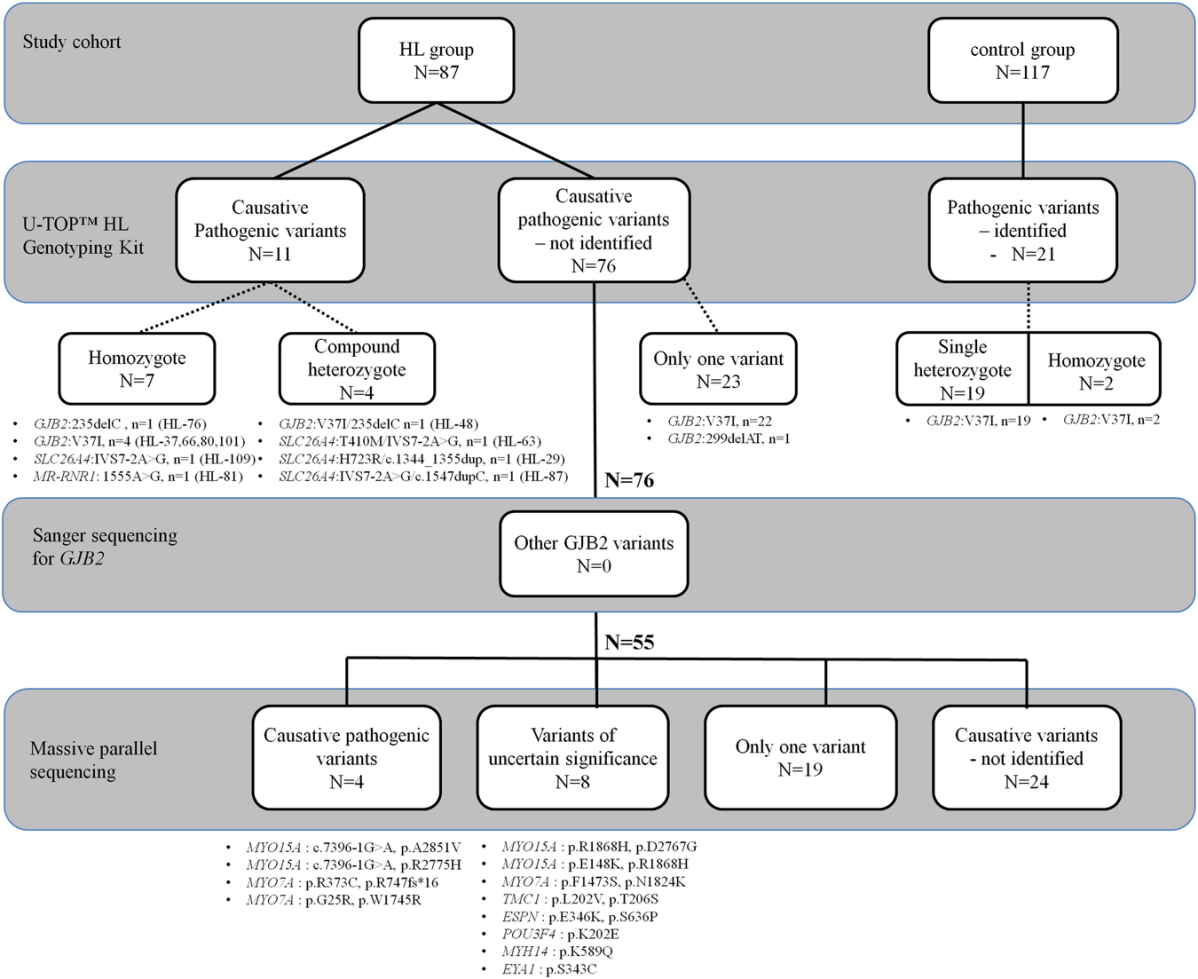
Overview of three-step protocol for genetic analysis. Our entire cohort comprises eighty-seven patients with severe-to-profound non-syndromic hearing loss (HL group) and 117 normal participants (control group). In the first step, a total of eleven subjects are identified to have causative pathogenic mutations. The second step of Sanger sequencing for *GJB2* does not additionally elucidate other *GJB2* pathogenic variants than the ones already screened through the first step. Next, massive parallel sequencing is performed for 55 patients of the HL group. Four patients with causative (likely) pathogenic variants and eight patients with variants of uncertain significance are identified.

To evaluate the genetic profile of our Vietnamese population for severe-to-profound NSHL, we utilized a three-step genetic analysis protocol **(**Fig. 1**)**. The first step was to screen for the 11 known deafness-causing variants using the U-TOP^TM^ HL Genotyping Kit. The second step was to perform Sanger sequencing of all the coding regions of *GJB2* in those who remained undiagnosed after the first step (N = 76). Finally, the third step involved massive parallel sequencing (MPS) in a subset of affected subjects (N = 55) that remained undiagnosed after the former two steps.

Among the 87 subjects segregating the severe-to-profound NSHL, complete molecular diagnosis was made on 11 subjects (N = 11/87, 12.6%) through the first screening step using the U_TOP HL genotyping kit **(**Fig. 1**)**. These eleven subjects were divided into two groups. The first group (N = 9) included those with causative variants detected by the screening genotyping kit—c.235delC of *GJB2* homozygotes (N = 1), p.V37I of *GJB2* homozygotes (N = 4), p.V37I/c.235delC of *GJB* compound heterozygote (N = 1), c.IVS7-2A > G of *SLC26A4* homozygotes (N = 1), p.T410M/c.IVS7-2A > G of *SLC26A4* compound heterozygote (N = 1), and c.1555 A > G of *MR-RNR1* (N = 1) **(**Fig. 1**)**. The second group (N = 2) included those who underwent Sanger sequencing of *SLC26A4* for further analysis—two compound heterozygotes of *SLC26A4:*p.H723R/c.1344_1355dup and c.IVS7-2A > G/c.1547dupC **(**Fig. 1**)**. To address the pathogenic potential of p.V37I in this population, the Vietnamese control group with normal hearing was also screened using U-TOPTM HL Genotyping Kit, with a special focus on p.V37I. A total of 19 single heterozygotes of p.V37I of *GJB2* and two homozygotes of p.V37I of *GJB2* were identified from the control group with normal hearing (N = 117) **(**Figs 1 and S1**)**. Resultantly, the frequency of p.V37I homozygote in the HL group (N = 4/87, 4.6%) was higher than that of the ethnicity-matched control group (N = 2/117, 1.7%), although the difference was not statistically significant (*p = *0.154).

All the remaining 76 subjects underwent the second step – Sanger sequencing of *GJB2* – to exclude other pathogenic variants, if any, elsewhere in *GJB2* **(**Fig. 1**)**. Single heterozygotes of p.V37I allele (N = 22) and single heterozygote of c.299delAT (N = 1) of *GJB2*, with no other pathogenic variants discovered elsewhere in *GJB2* through *GJB2* Sanger sequencing, were considered undiagnosed in the HL group **(**Fig. 1**)**. None of these subjects carried the known non-coding region variants of *GJB2* or the known large deletions of *GJB6*.

To further elucidate the causative variants in subjects who remained undiagnosed after the first two steps, the third step – MPS targeting the genes related to hearing loss – was performed **(**Fig. 1**)**. Among the 55 subjects, four (7.3%) subjects turned out to have ‘causative pathogenic variants’ involving two genes (*MYO15A*, *MYO7A*). **(**Fig. 1 and Table 1**)**. Meanwhile, eight subjects (N = 8/55, 14.5%) with severe-to-profound NSHL were classified as the group with variants of uncertain significance. Five subjects with variants in *MYO15A*, *MYO7A*, *TMC1*, and *ESPN* genes, which were inherited in autosomal recessive mode, were identified. In addition, one boy (HL-78) with a single novel variant of uncertain significance in *POU3F4* with X-linked inheritance pattern and two subjects with a single variant in *MYH14* and *EYA1* genes with autosomal dominant inheritance pattern were identified (Fig. 1 and Table 1). In contrast, 19 subjects had only one variant with pathogenic potential or uncertain significance (34.5%) in the autosomal recessive deafness genes (see Supplementary Table S1**)** without reaching a final molecular genetic diagnosis, and the other 24 subjects (43.6%) did not carry any potentially pathogenic variant as documented by MPS **(**Fig. 1**)**. In this study, the overall molecular diagnostic rate through our three-step protocol in our Vietnamese population with severe-to-profound NSHL was estimated to be 31.7% when the subjects with variants of uncertain significance were included and 19.0% when they were not.

**Table 1:** Details of twelve deaf subjects having causative pathogenic variants or variants of uncertain significance after massive parallel sequencing targeting the genes related with hearing loss.

Gene (GeneBank No.)	Family ID	Variant	Classification of variants	State	Depth (DP/AD)	Q call (Qual/MQ)	Prediction Algorithm			Conservation Score		MAF		Published reference (PMID)
							Mutation Taster	PolyPhen-2	SIFT	PhyloP	GERP + +	ExAC, 1000 G	GnomAD*	
**Causative (likely) pathogenic variants**														
*MYO15A* (NM_016239)	HL-92	c.7396-1 G > A	LPa	Het	61	60	DC	NA	NA	4.783	4.01	A = 0.00002/1 (ExAC)	A = 0.0001 (2/14966)	This study
	HL-92	c.8552 C > T:p.Ala2851Val	Pa	Het	80	60	DC	PrD	D	5.319	4.41	T = 0.000008/1 (ExAC)	T = 0.000065, (1/15292)	This study
	HL-105	c.7396-1 G > A	LPa	Het	85	60	DC	NA	NA	4.783	4.01	A = 0.00002/1 (ExAC)	A = 0.0001 (2/14966)	This study
	HL-105	c.8324 G > A:p.Arg2775His	Pa	Het	123	60	DC	PrD	D	5.89	5.1	T = 0.000008/1 (ExAC)	T = 0.000 (0/245848)	23767834, This study
*MYO7A* (NM_000260)	HL-110	c.1117 C > T:p.Arg373Cys	Pa	Het	125	60	DC	PrD	D	4.201	5.11	ND	ND	22903915, This study
	HL-110	c.2239_2240delAG: p.Arg747fs*16	Pa	Het	65	60	NA	NA	NA	1.305_ 3.153	2.7	ND	Del = 0.00002 (1/33480)	22898263, This study
	HL-49	c.73 G > A:p.Gly25Arg	Pa	Het	392	60	DC	PrD	D	5.733	4.69	A = 0.00002/2 (ExAC)	A = 0.000018 (2/110120)	9002678, This study
	HL-49	c.5233 T > G:p.Trp1745Arg	US	Het	104	60	DC	PrD	D	1.028	5.13	ND	ND	This study
**Variants of uncertain significance**														
*MYO15A* (NM_016239)	HL-108	c.8300 A > G:p.Asp2767Gly	US	Het	53	60	DC	PrD	D	4.864	5.1	ND	ND	This study
	HL-108	c.5603 G > A:p.Arg1868His	US	Het	62	60	DC	PrD	T	3.273	4.8	A = 0.0001/15 (ExAC) A = 0.0004/2 (1000 G)	A = 0.0005 (11/18868)	This study
	HL-79	c.442 G > A:p.Glu148Lys	US	Het	195	60	P	B	T	1.087	5.25	ND	ND	This study
	HL-79	c.5603 G > A:p.Arg1868His	US	Het	55	60	DC	PrD	T	1.048	4.8	A = 0.0001/15 (ExAC) A = 0.0004/2 (1000 G)	A = 0.0005 (11/18868)	This study
*MYO7A* (NM_000260)	HL-47	c.4418 T > C:p.Phe1473Ser	US	Het	257	60	DC	PrD	D	4.693	5.5	ND	ND	This study
	HL-47	c.5472 C > G:p.Asn1824Lys	US	Het	122	60	DC	PrD	D	1.98	3.49	ND	ND	This study
*TMC1* (NM_138691)	HL-44	c.604 C > G: p.Leu202Val	US	Het	112	60	DC	PrD	T	1.871	5.78	ND	ND	This study
	HL-44	c.616 A > T:p.Thr206Ser	US	Het	110	60	DC	PrD	T	5.038	5.78	ND	ND	This study
*ESPN (*NM_031475)	HL-106	c.1036 G > A:p.Glu346Lys	US	Het	707	60	DC	PrD	D	4.523	3.77	A = 0.00007/9 (ExAC) A = 0.0002/1 (1000 G)	A = 0.00009 (17/18870)	This study
	HL-106	c.1906T > C:p.Ser636Pro	US	Het	23	24.8	P	B	D	1.289	5.1	ND	ND	This study
*POU3F4 (*NM_000307)	HL-78	c.604 A > G:p.Lys202Glu	US	Hemi	86	60	D	PrD	NA	4.676	5.31	ND	ND	This study
*MYH14 (*NM_001145809)	HL-70	c.1765A > C:p.Lys589Gln	US	Het	270	60	DC	PrD	D	4.091	4.09	C = 0.00002/2 (ExAC)	C = 0.000017, (2/111562)	This study
*EYA1 (NM_000503)*	HL-34	c.1028 C > G: p.Ser343Cys	US	Het	(81/29)	(709/60)	DC	PrD	D	5.842	5.86	ND	ND	This study

^*^Maximum minor allele frequency among all populations in gnomAD; DP, total depth; AD, alternative allele depth; Qual, SNP quality; MQ, mapping quality; LPa, likely pathogenic; Pa, pathogenic; US, uncertain significance; Het, Heterozygous; Hom, Homozygous; Hemi, Hemizygous; P, Polymorphism; DC, Disease causing; PrD, Probably damaging; PsD, Possibly damaging; D, Damaging; B, Benign; T, Tolerated; ND, not detected; NA, not applicable; PMID, PubMed ID (PMID is the unique identifier number used in PubMed.; PhyloP score from the Mutation Taster (http://www.mutationtaster.org/); in silico prediction Algorithm: Polyphen-2 (http://genetics.bwh.harvard.edu/pph2/index.shtml); SIFT (http://sift.jcvi.org/www/SIFT_chr_coords_submit.html); Conservation tools: GERP +  + score in the UCSC Genome Browser (http://genome-asia.ucsc.edu/); ExAC, Exome Aggregation Consortium (http://exac.broadinstitute.org/); 1000 Genomes (https://www.ncbi.nlm.nih.gov/variation/tools/1000genomes/); GO-ESP, NHLBI GO Exome Sequencing Project (http://evs.gs.washington.edu/EVS/); GnomAD, genome Aggregation Database (http://gnomad.broadinstitute.org/).

### Mutation spectrum in Vietnamese population

The *MYO15A* variants were the most common cause of severe-to-profound NSHL in our Vietnamese population, and the prevalence of the *MYO15A* variants that were classified as (likely) pathogenic variants or uncertain significance was 7.2% (N = 4/55) **(**Table 2**)**. Next, the autosomal recessive variants of *GJB2* (6.9%, N = 6/87), *MYO7A* (5.5%, N = 3/55), and *SLC26A4* (4.6%, N = 4/87) were identified as the next tier of causes in this population **(**Table 2**)**. The frequency of variants in *ESPN* (1.8%, N = 1/55), *TMC1* (1.8%, N = 1/55), *POU3F4* (1.8%, N = 1/55), *MYH14* (1.8%, N = 1/55), and *MR-RNR1* (1.1%, N = 1/87) were less common in our Vietnamese population with severe or greater degree of HL.

**Table 2:** Mutation spectrum of severe-to-profound non-syndromic hearing loss in Vietnamese pediatric population.

Gene	Mode of inheritance	Causative pathogenic variants^†^	Variants of uncertain significance^†^	Prevalence (%)
*MYO15A*	AR	2 (3.6%)	2 (3.6%)	7.2
*GJB2*	AR	6 (6.9%)*		6.9
*MYO7A*	AR	2 (3.6%)	1 (1.8%)	5.5
*SLC26A4*	AR	4 (4.6%)		4.6
*TMC1*	AR		1 (1.8%)	1.8
*ESPN*	AR		1 (1.8%)	1.8
*POU3F4*	X-lined		1 (1.8%)	1.8
*MYH14*	AD		1 (1.8%)	1.8
*EYA1*	AD		1 (1.8%)	1.8
*MR-RNR1*	mitochondrial	1 (1.1%)		1.1

^†^Number of diagnosis (prevalence, %); *, the homozygous p.V37I variants of *GJB2* gene were included; AR, autosomal recessive; AD, autosomal dominant; No, number.

## Discussion

In this study, we elucidated the mutation spectrum of severe-to-profound NSHL in the Vietnamese pediatric population for the first time. *MYO15A*, *GJB2*, *MYO7A*, and *SLC26A4* variants were shown to be the leading causes in our cohort, and the estimated prevalence was 7.2%, 6.9%, 5.5%, and 4.6%, respectively. The variants of *TMC1*, *ESPN*, *POU3F4*, *MYH14*, *EYA1*, *and MR-RNR1* genes followed those from the top tier deafness genes in the order of frequency as a molecular etiology of severe-to-profound NSHL in Vietnamese.

It may be worth noting that the mutation spectrum of this Vietnamese deaf population is unique and distinct. First, there was a significantly lower genetic load of *GJB2* variants compared with other ethnic populations, including East Asians. The prevalence of the DFNB1 due to *GJB2* variants in our Vietnamese pediatric population with severe-to-profound NSHL was calculated to be 6.9%, which could be further lowered to 2.3% if the p.V37I homozygotes were excluded. *GJB2* variants are the leading genetic etiology of NSHL in many populations, even though the detection rate of *GJB2* variants can significantly differ depending on the ethnicity and country. The prevalence of *GJB2*-associated hearing loss was quite variable, ranging from 0% in South Africa to 40~50% in Eastern Europe^25^. According to a nationwide genetic study about the *GJB2* mutation spectrum in China, there was a significant difference in the detection rate of pathogenic *GJB2* allele depending on the region within China, varying from 4% in Guangxi to 30.4% in Jiangsu, while the overall frequency of pathogenic *GJB2* allele among moderate to severe hearing loss was 17.9%^26^. Interestingly, Guangxi, a city associated with a low prevalence of *GJB2* variants, is in the southern part of China, bordering Vietnam. Vietnam is the easternmost country on the Indochina Peninsula, bordering China to the north, Laos to the northwest, Cambodia to the southwest, and Thailand across the Gulf of Thailand to the southwest. The population of Vietnam was approximately 94.6 million in 2016, and the ethnic group, “Kinh,” comprised 86% of the total population. Kinh has been known to originate from northern Vietnam. Recent phylogenetic studies revealed that the majority of Kinh originated from South China and a minority from Thailand and Indonesia^27^. Therefore, the low prevalence of detectable *GJB2* variants could be viewed as the genetic similarity between the Kinh people and the people from southern parts of China. This suggests a close ethnic relationship between these groups. Low prevalence of detectable *GJB2* variants does not necessarily indicate low prevalence of DFNB1 in these populations and might suggest the presence of unidentified variants, such as large genomic deletions residing somewhere in the DFNB1 locus, and thus affecting *GJB2* expression and function. These variants would have evaded our current molecular diagnostic protocol. This hypothesis requires future confirmation. The genetic similarity with the southern parts of China can also be seen in the *SLC26A4* mutation. Unlike the northern area of China, in which the prevalence of *SLC26A4* mutation has been reported to range from 10 to 13%^28–30^, patients with severe-to-profound hearing from the Guangxi province showed limited prevalence of *SLC26A4* variants (2.2%)^31^, similar to our Vietnamese cohort.

Second, the detection frequency of p.V37I in our Vietnamese population with severe-to-profound NSHL (18.4%) was significantly higher than that in East Asian populations (1~6.7%)^26,32,33^ and European populations (0.6%)^25^. This allele was also frequently detected in the population of Thailand, which is bordered by Vietnam (16.7%)^34^, questioning the causative role of this allele to severe to profound deafness in Vietnamese. Among the 23 subjects in the HL group with one p.V37I allele detected on the first screening step, four subjects had pathogenic or uncertain significance variants in other deafness genes unrelated to *GJB2* (HL-63, *SLC26A4*:T410M/IVS7-2A > G; HL-49, *MYO7A*: p.G25R/ p.W1745R; HL-34, *EYA1*:p.S343C; HL-44, *TMC1*: p.L202V/p.T206S) **(**Table 1**)**. The other 19 subjects showed either one (N = 10, HL-35, 46, 53, 57, 60, 72, 84, 86, 97, 99) or no (N = 9, HL-22, 27, 30, 33, 43, 67, 68, 69, 98) variant with pathogenic potential from other unrelated deafness genes (see Supplementary Table S1**)**. Interpretation of causality between p.V37I homozygotes and severe-to-profound deafness warrants further caution because two homozygotes of p.V37I of *GJB2* were also identified in the control group with normal hearing **(**Figs 1 and S1**)**, and the prevalence of the homozygotes of p.V37I of *GJB2* (1.7%) in the control group was not significantly different from that (4.6%) in the HL group (*p* = 0.154). However, the p.V37I variant has been previously reported to have a diverse clinical presentation, ranging from normal-to-profound hearing loss^35,36^, and the prevalence of p.V37I homozygote was, even if not statistically significant, higher in the HL group than in the control group (4.6% vs 1.7%) in our study. Given this, at least a subset of p.V37I homozygotes in the Vietnamese population can potentially cause severe-to-profound hearing loss. Both normal hearing from the p.V37I homozygotes in the control group and profound hearing loss from some p.V37I homozygotes in the HL group might be phenotypes of both extremes from the same genetic alteration, which is modulated by modifiers. To clarify the pathogenic role of p.V37I in severe-to-profound hearing loss, further study evaluating the influence of genetic modifiers on the phenotype of homozygous p.V37I variant or exploring the yet-to-be identified, pathogenic DFNB1 allele *in trans* with single heterozygous p.V37I, such as copy number variation (CNV)^17,37–40^, is warranted.

Third, *MYO15A* was shown to be the most frequent causative gene (7.2%) in our Vietnamese population, with a much higher prevalence than that in the European populations (3.1%)^41^. In fact, the *MYO15A* gene, which is a causative gene of DFNB3 (OMIM 600316) and has been known to be associated with moderate to severe hearing loss as well as profound hearing loss, is usually ranked as the third or fourth most common cause of DFNB deafness in many ethnicities^42–45^. The *MYO7A* variants also accounted for a significant proportion (5.5%) of severe-to-profound hearing loss in our Vietnamese population, which was higher than the estimates found in other studies^42–44^. *MYO7A* is located on 11p13.5, encoding the myosin 7 A protein. To date, there have been more than 280 different pathogenic or likely pathogenic mutations and 380 variants of uncertain significance reported from *MYO7A* (https://www.ncbi.nlm.nih.gov/clinvar as of Aug 2018), which have been associated with Usher syndrome 1B^46^, non-syndromic autosomal recessive hearing loss (DFNB2)^47^, and autosomal dominant hearing loss (DFNA11)^48^. Patients diagnosed with Usher syndrome type 1B having *MYO7A* variants typically show profound hearing loss at birth and progressive retinal degeneration^49^.

Another interesting finding is that the potentially pathogenic autosomal dominant variant of *MYH14* was considered as a contributor to severe-to-profound hearing loss in our Vietnamese population. The *MYH14* gene encodes myosin-14 protein and was reported to be an important causative gene for prelingual severe NSHL with autosomal dominant inheritance. This is unique considering the usual auditory phenotype of autosomal dominant deafness genes^50^. In our present study, we were unable to determine whether this *MYH14* variant occurred *de novo* due to the lack of parental samples, even though auditory phenotypes of parents were allegedly normal. Therefore, we classified these as variants of uncertain significance. Nonetheless, the *MYH14* variant was still considered the variant with pathogenic potential due to their extremely rare MAF in publicly available databases.

Currently, the diagnostic yield from the first two steps – U-TOP^TM^ HL genotyping kit and Sanger sequencing of *GJB2*, respectively – does not seem high, only reaching 12.6% including the homozygous p.V37I variants of *GJB2*. The U-TOP^TM^ HL genotyping kit, initially designed to screen East Asian subjects with hearing loss, does not appear to be suitable for the Vietnamese population with hearing loss, where *MYO15A* and *MYO7A* were the most frequent causative genes. Accordingly, an efficient screening kit customized for the mutation spectrum of this population should be designed. Moreover, we were able to detect the causative pathogenic variants or variants of uncertain significance using MPS in about 21.8% (N = 12/55) of our subjects with NSHL. In total, the diagnostic yield of our three-step molecular diagnostic protocol, including MPS, was estimated to be 31.7% in our Vietnamese population, which was inferior to other studies that used massively parallel sequencing^3,18,22,41,51^.

There were several limitations in this study, with respect to the confirmation of pathogenicity of detected variants and diagnostic rate of our molecular diagnostic protocol. We were unable to perform additional analyses, including a segregation study necessary to confirm the co-segregation of the variants with deafness among the family members and the *trans*-configuration of two compound heterozygous due to lack of parental samples in our cohort. Therefore, some genotypes obtained from the MPS analysis may not be conclusive, and the diagnostic yield of the three-step protocol employed in our study for molecular diagnosis of severe-to-profound hearing loss may be slightly overestimated. These issues could be addressed in a future study using segregation analysis or MPS with the inclusion of family members. Secondly, the variants of other deafness genes or CNVs, which were not included in the targeted deafness genes in this study or could not be detected with our diagnostic protocol, may have been missed. CNV has been identified as one of the frequent causes of ARNSHL. According to the literature, at least one CNV in a known deafness gene was found in 15.2% of patients with hearing loss, and causative CNVs were identified in 7.3% of these patients as homozygous, hemizygous in conjunction with a second pathogenic mutation, or biallelic CNVs^37^. Large genomic deletions, involving *GJB2* or GJB6, have previously been reported as a causative mutation related to hearing loss^38–40^. Therefore, novel or unrevealed CNVs in the coding or noncoding region of *GJB2* or *GJB6*, which would lead to null function, can explain severe-to-profound auditory phenotypes in our participants in a *trans* configuration with the single heterozygous p.V37I (N = 19/87, 21.8%). As aforementioned, the prevalence of *GJB2* variants in our Vietnamese population was estimated to be only 2.3% when the p.V37I homozygotes were excluded. However, upon discovery of such occult genomic deletion, some of the single heterozygotes of p.V37I can later be classified as DFNB1, leading to an increase in the prevalence of DFNB1 in the Vietnamese population. Hence, in the next study, the presence of occult genomic deletion, if any, in the DFNB1 locus (either DFNB1A or DFNB1B) among these putative p.V37I carriers in a Vietnamese population will be explored by multiple ligation probe amplification (MLPA) covering the entire DFNB1 locus. Lastly, MPS test was not performed in 25 patients and their genetic diagnosis remained inconclusive, which may have been another limitation of this study.

In conclusion, our study is the first attempt to reveal the characteristics of mutation spectrum of severe-to-profound NSHL in the Vietnamese population, providing the basis for the establishment of a comprehensive molecular genetic diagnostic protocol. Furthermore, our data provide supportive evidence suggesting an ethnic relationship between the peoples of Vietnam and those in the southern parts of China.

## Methods

### Participants

Between September 2015 and May 2016, children who visited the Children Hospital 1 in Vietnam with delayed language development or decreased responses to sound stimuli were included in this study. A total of 87 children (48 males, 39 females) were selected and included. All participants underwent an audiological evaluation. The hearing thresholds at 0.5, 1, 2, and 4 kHz were evaluated with pure-tone audiometry, and the average threshold was calculated. In the cases where pure-tone audiometry could not be performed due to the subjects’ young age, the hearing level was determined using the auditory brainstem response threshold test or visual reinforcement audiometry. The inclusion criteria were severe-to-profound hearing loss with over 70 dB of hearing thresholds. Children who had syndromic hearing loss or familial history of hearing loss were excluded; to avoid any bias from acquired hearing loss, subjects who were older than 12 years were also excluded. The mean age was 4.6 ± 2.2 (0–11) years at the time of their initial visitation. Moreover, a total of 117 participants (40 males, 77 females) with normal hearing under 25 dB were included in the control group. The mean age of the control group was 33.5 ± 9.6 (20–66) years. The Institutional Review Boards (IRBs) of Seoul National University Bundang Hospital (SNUBH) in Korea (IRB-B-1007-105-402) and Research Center of Children Hospital 1 in Vietnam (CS/1/15/13) approved this study. All procedures were performed in accordance with relevant guidelines and regulations. We obtained written informed consent from all participants in accordance with the Declaration of Helsinki; in the case of children, written informed consent was obtained from their patient or guardian. After obtaining written informed consent, ten milliliters of whole blood for genetic analysis was obtained from all participants. All data generated or analyzed during this study are included in this published article and its supplementary information file.

### U-TOP screening test

A total of 87 subjects in the HL group and 117 normal participants in the control group of our Vietnamese population were screened using the U-TOP™ HL Genotyping Kit (SeaSun Biomaterials, Daejeon, Korea), which is a real-time PCR-based MeltingArray genetic diagnostic kit **(**Fig. 1**)**. This new screening tool was designed to detect variants known to be associated with mild-to-moderate hearing loss, as well as to screen variants associated with severe-to-profound hearing loss^52^. The molecular diagnostic platform, based on ethnicity-specific mutation spectrums of sensorineural hearing loss, contain 11 variants of 5 genes (*GJB2*: p.V37I, c.235delC, c.299delAT, p.R143W; *SLC26A4*: p.H723R, c.IVS7-2A > G, p.T410M, p.L676Q; *MTRNR1*: 1555 A > G; *TMPRSS3*: p.A306T; *CDH23*: p.P240L) showing high prevalence with varying degrees in the Korean population. The phenotype of the p.V37I variant of *GJB2* was reported as mild to profound hearing loss^53–55^. Therefore, the homozygosity of p.V37I allele of *GJB2* was considered as causative pathogenic variant in this study. The compound heterozygote of V37I and other pathogenic variant of *GJB2*, such as p.R143W, were proposed as possible causative variant of moderate to severe hearing loss^55^, and were included as causative pathogenic variant in this study.

When only one variant of either *GJB2* or *SLC26A4* was detected with the screening kit, Sanger sequencing for the entire coding region of the corresponding gene, beyond the coverage of the screening kit, was additionally performed to find the second variant. A rare novel variant of *SLC26A4* in a *trans* configuration with a definitely pathogenic *SLC26A4* variant was considered as ‘pathogenic’, as previously proposed^56^.

### Sanger sequencing for *GJB2*

For 76 subjects whose causative genetic variants were not identified with a screening test, Sanger sequencing for the coding region of the *GJB2* gene was performed **(**Fig. 1**)**. Next, Sanger sequencing for previously reported, non-coding variants of *GJB2* and evaluation of known genomic deletion of *GJB6* was performed. In detail, Sanger sequencing for the four known pathogenic variant on the noncoding regions, c.-22-2A > C^57^, c.-23G > T^58^, c.−23 + 1 G > A^59^, and c.−259C > T^60^ was performed with two primer sets, as previously described^61^. Next, a multiplex breakpoint PCR assay was performed for two previously reported large genomic deletions (del[*GJB6*-D13S1830] and del[*GJB6*-D13S1854])^40^. To detect other structural variations on the 5-kb regions upstream of *GJB2* within the DFNB1 locus, we also verified the raw data of MPS, if available, using the Integrative Genomic Viewer (http://www.broadinstitute.org/igv/home).

### Massive parallel sequencing

A subset of subjects whose causal genetic variants were not identified with the U-TOPTM HL genotyping kit were evaluated with targeted exome sequencing (TES; Otogenetics, Norcross, GA, USA) and whole exome sequencing (WES; Otogenetics, Norcross, GA, USA). TES and WES were captured using the NimbleGen Sequence Catcher (Roche NimbleGen Inc., Madison, WI, USA) and SureSelect 50 Mb Hybridization and Capture kit, respectively. Bioinformatics analyses were performed, as previously described, against the targeted genes related to hearing loss **(**Fig. 1**)**^62,63^. In total, 32–69 million short reads (100-bp paired-end reads) were obtained via MPS. More than 85% of the target exon regions were covered by at least five sequence reads. The reads were mapped onto the UCSC hg19 reference genome. The non-synonymous single nucleotide polymorphisms (SNPs) were filtered with a depth ≥ 15. Minor allele frequency (MAF) of the variants was evaluated from publicly available databases, including ExAC (http://exac.broadinstitute.org/), 1000 Genomes (https://www.ncbi.nlm.nih.gov/variation/tools/1000genome), GO-ESP (http://evs.gs.washington.edu/EVS/), and GnomAD (http://gnomad.broadinstitute.org/). Variants with MAF ≥ 0.5% were excluded, unless they were pathogenic according to the literature, ClinVar, or Deafness Variation Database (http://deafnessvariationdatabase.org/) (Accessed: May 26, 2018)^64^. We used a threshold of 0.5% for ARNSHL and 0.05% for ADNSHL^65^. We interpreted and classified the pathogenicity of our variants as one of the followings: Pathogenic, likely pathogenic, benign, likely benign, and uncertain significance according to the ACMG guidelines (Table 1 and Supplementary Table S1)^66^. Patient cohort was divided into four groups: (1) patients with causative (likely) pathogenic variants; (2) patients with variants of uncertain significance; (3) patients with only one variant of the gene with autosomal recessive inheritance pattern; (4) patients without detected causative variants **(**Fig. 1**)**^22^.

## Supplementary information

Supplementary information
