# Supplementary material for: Elucidation of the unique mutation spectrum of severe hearing loss in a Vietnamese pediatric population
Source: Sci Rep. 2019 Feb 7;9:1604. doi: 10.1038/s41598-018-38245-4 (PMC6367484; doi:10.1038/s41598-018-38245-4)
Supplement: Supplementary file 1 — Supplementary information [file 41598_2018_38245_MOESM1_ESM.pdf]

Supplementary information

Title: Elucidation of the unique mutation spectrum of severe hearing loss in a Vietnamese pediatric population

Jae Joon Han[1+], Pham Dinh Nguyen[2+], Doo-Yi Oh[1], Jin Hee Han[1], Ah-Reum Kim[3], Min Young Kim[1], Hye-Rim Park[1], Lam Huyen Tran[4], Nguyen Huu Dung[4], Ja-Won Koo[1], Jun Ho Lee[5], Seung Ha Oh[5], Hoang Anh Vu[6&], Byung Yoon Choi[1*&]

1. Department of Otorhinolaryngology-Head and Neck Surgery, Seoul National University Bundan g Hospital, Seongnam, Korea
2. Department of Otorhinolaryngology, Children's Hospital 1, Ho Chi Minh City, Vietnam
3. Biomedical Research Institute, Seoul National University Hospital, Seoul, Korea
4. Department of Otorhinolaryngology, University of Medicine and Pharmacy at Ho Chi Minh Ci ty, Vietnam.
5. Department of Otorhinolaryngology-Head and Neck Surgery, Seoul National University Hospita l, Seoul, Korea
6. Center for Molecular Biomedicine, University of Medicine and Pharmacy at Ho Chi Minh Cit y, Vietnam.

***Corresponding authors:**

Byung Yoon Choi (email: choiby@snubh.org)

Hoang Anh Vu (email: hoangvuxinh@yahoo.com).

[+]These authors contributed equally to this work.

[&]These authors also supervised this work equally.

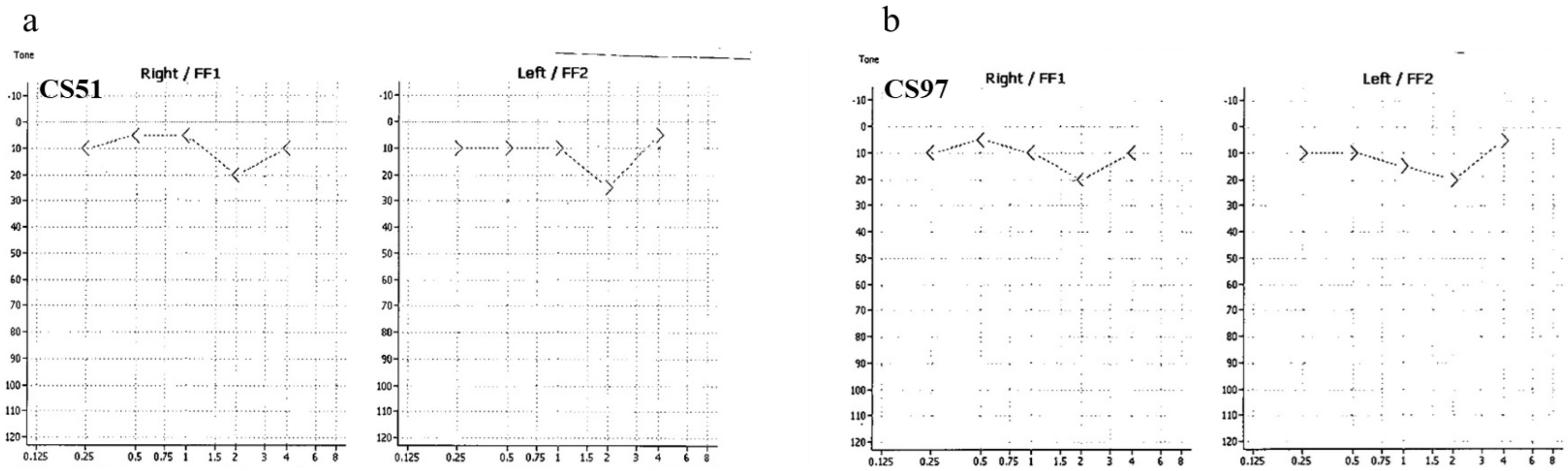

Supplementary Figure S1. Audiograms of the two control participants carrying homozygous p.V37I variant of GJB2 gene. CS51(a, 32-year old female) and CS97 (b, 36-year old male) who were enrolled in control group showed normal bone conduction hearing thresholds on both sides within 25 dB. Using U-TOPTM HL Genotyping Kit and Sanger sequencing for GJB2 gene, homozygotes of p.V37I of GJB2 were identified in the two participants of control group.

Supplementary Table S1. Details of the subjects having only one causal variant after massive parallel sequencing targeting the genes related with hearing loss.

Family ID	Gene (GeneBank No.)	Variant		State	Depth (DP/ AD)	Q call (Qual/ MQ)	Prediction Algorithm			Conservation Score		MAF		Published reference (PMID)
							Mutation Taster	PolyPhen-2	SIFT	PhyloP	GERP++	ExAC, 1000G, GO-ESP	GnomAD*	
HL-33	*ESPN* (NM_031475)	c.2557A>G:p.Lys853Glu	US	Het	245	60	DC	PrD	D	3.759	4.32	G=0.00002/2 (ExAC) G=0.0002/1 (1000 G)	ND	This study
	MYO3A (NM_017433)	c.544C>T:p.Arg182Trp	US	Het			DC	PrD	D	2.928	4.75	T=0.00004/5 (ExAC)	T=0.00037 (7/18860)	This study
HL-46	*TSPEAR*(NM_144991)	c.607C>T:p.Arg203Trp	US	Het	(67/29)	(836/60)	DC	PrD	D	-0.018	-0.028	A=0.0001/13 (ExAC), A=0.0008/4 (1000 Genomes), A=0.00008/1 (GO-ESP)	A=0.0005 (15/30754)	This study
HL-53	*USH2A (NM_206933)*	c.6929C>T:p.Thr2310Met	US	Het	(56/25)	(741/60)	DC	PrD	D	4.892	5.81	A=0.00008/10 (ExAC) A=0.0006/3 (1000 Genomes) A=0.00008/1 (GO-ESP)	A=0.0003 (6/18848)	This study
	PDZD7(NM_001195263)	c.2239C>T:p.Arg747Trp	US	Het	(39/24)	(952/60)	P	PsD	D	-0.276	-0.995	ND	ND	This study
HL-57	*GPR98* (NM_032119)	c.16447T>C:p.Phe5483Leu	US	Het	(71/35)	(1050/60)	DC	PrD	D	2.557	4.56	C=0.00010/12 (ExAC) C=0.0006/3 (1000 Genomes)	C=0.0016 (31/18864)	This study
	CDH23 (NM_022124)	c.6376C>T:p.Arg2126Cys	US	Het	(206/101)	(2974/60)	DC	PrD	D	0.932	4.61	C=0.00010/12 (ExAC) C=0.0006/3 (1000 Genomes)	C=0.00035 (6/17246)	This study
HL-60	*USH2A (NM_206933)*	c.10859T>C:p.Ile3620Thr	Pa	Het	(139/69)	(1980/60)	DC	D	D	4.091	5.9	G=0.000008/1 (ExAC)	G=0.0001 (2/17248)	25268133, This study
HL-67	*TECTA* (NM_005422)	c.5472G>A:p.Gly1824Asp	Pa	Het	134	60	DC	PrD	D	5.939	6.05	A=0.00004/5 (ExAC)	A=0.00058 (11/18870)	9590290, 20301607,

														24363064, This study
HL-72	*MYO7A* (NM_000260)	c.6092G>A:p.Arg2031Gln	US	Het	(121/65)	(2052/60)	DC	PsD	T	1.048	3.7	A=0.00006/2 (ExAC)	ND	This study
HL-80	*MYO15A (NM_016239)*	c.7730A>T p.Lys2577Met	US	Het	(142/69)	(2033/60)	DC	PrD	D	0.703	2.91	ND	ND	This study
	TECTA (NM_005422)	c.5000A>G p.Glu1667Gly	US	Het	(83/38)	(1031/60)	DC	PsD	D	4.861	5.54	G=0.00002/3 (ExAC)	G=0.00068 (13/18868)	This study
HL-84	*ILDR1 (NM_001199799)*	c.1578A>T p.Lys526Asn	US	Het	(93/43)	(1132/60)	DC	PsD	D	0.547	2.09	A=0.00002/1 (ExAC)	ND	This study
HL-86	*PEX1 (NM_000466)*	c.2966T>C p.Ile989Thr	Pa	Het	(46/19)	(659/60)	DC	PrD	D	5.127	5.74	G=0.00005/6 (ExAC) G=0.0004/2 (1000 Genomes)	G=0.001 (19/18856)	16088892, This study
HL-88	*MYO15A* (NM_016239)	c.4156G>A:p.Gly1386Ser	US	Het	178	60	DC	PrD	D	5.775	4.78	ND	ND	This study
HL-91	*BSND (*NM_057176)	c.88C>T: p.Arg30Trp	US	Het	362	60	P	PrD	D	1.103	3.51	T=0.000008/1 (ExAC)	T=0.00004 (1/24022)	This study
	*MYO3A (*NM_017433)	c.1325A>G:p.His442Arg	US	Het	144	60	DC	PrD	D	5.147	5.79	G=0.00005/6 (ExAC)	G=0.00037 (7/18862)	This study
HL-94	*GJB3 (*NM_024009)	c.580G>A:p.Ala194Thr	US	Het	704	60	DC	B	T	3.045	4.16	A=0.0009/107 (ExAC) A=0.0014/7 (1000 G)	A=0.007 (132/18862)	19050930, This study
HL-97	*PEX6*	c.635T>A p.Leu212His	US	Het	(137/75)	(1993/60)	DC	PrD	D	2.73	4.73	T=0.00002/2 (ExAC)	T=0.0002 (4/17240)	This study
	USH2A	c.14218G>A p.Ala4740Thr	LPa	Het	(83/38)	(1028/60)	DC	B	D	1.166	4.82	ND	A=0.0001 (2/17246)	This study
HL-99	*MYO15A (NM_016239)*	c.8910_8939del: p.Val2971_Ala2980del	US	Het	(33/7)	(202/60)	NA	NA	NA	NA	NA	del=0.0001/10 (ExAC)	del=0.0015 (25/16198)	This study
HL-100	*PJVK (*NM_001042702)	c.387A>C:p.Leu129Phe	US	Het	87	60	DC	PrD	D	-0.064	0.88	ND	ND	This study
HL-101	*CDH23 (NM_022124)*	c.611C>T p.Thr204Met	US	Het	(133/75)	(2001/60)	DC	PrD	D	1.819	3.48	T=0.00002/2 (ExAC)	T=0.00003 (1/30764)	This study
HL-103	*KCNJ10 (*NM_002241)	c.1042C>T:p.Arg348Cys	Pa	Het	336	60	DC	PrD	D	1.048	4.18	A=0.0002/29 (ExAC) A=0.0006/3 (1000 G) A=0.00008/1 (GO-ESP)	A=0.0018 (34/18866)	20301607, 20301640, 19426954, This study
	*SLC26A5 (*NM_198999)	c.28C>T:p.Leu10Phe	US	Het	383	60	DC	PsD	T	3.947	5.27	ND	A=0.00006 (2/33558)	This study

HL-104	*ESRRB* (NM_004452)	c.520C>T:p.Arg174Cys	US	Het	306	60	DC	PrD	D	5.892	4.92	A=0.00008/1 (GO-ESP)	ND	This study
	MYO15A (NM_016239)	c.4139A>G:p.Glu1380Gly	US	Het	179	60	DC	PrD	D	4.836	5.22	G=0.0002/1 (1000 G)	ND	This study
HL-111	*BSND* (NM_057176)	c.10G>A:p.Glu4Lys	US	Het	307	60	DC	PrD	D	3.067	4.44	A=0.00007/8 (ExAC) A=0.0002/1 (1000 G)	A=0.0006 (11/17248)	This study
HL-114	*WHRN* (NM_015404)	c.2492A>G:p.Glu831Gly	US	Het	80	60	DC	PrD	D	4.96	4.96	C=0.000008/1 (ExAC)	C=0.000057 (1/17244)	This study

*, maximum minor allele frequency among all populations in gnomAD; DP, total depth; AD, alternative allele depth; Qual, SNP quality; MQ, mapping quality; LPa, likely pathogenic; Pa, pathogenic; US, uncertain significance; Het, Heterozygous; Hom, Homozygous; Hemi, Hemizygous; P, Polymorphism; DC, Disease causing; PrD, Probably damaging; PsD, Possibly damaging; D, Damaging; B, Benign; T, Tolerated; ND, not detected; NA, not applicable; PMID, PubMed ID (PMID is the unique identifier number used in PubMed.; PhyloP score from the Mutation Taster (http://www.mutationtaster.org/); in silico prediction Algorithm: Polyphen-2 (http://genetics.bwh.harvard.edu/pph2/index.shtml); SIFT (http://sift.jcvi.org/www/SIFT_chr_coords_submit.html); Conservation tools: GERP++ score in the UCSC Genome Browser (http://genome-asia.ucsc.edu/); ExAC, Exome Aggregation Consortium (http://exac.broadinstitute.org/); 1000 Genomes (https://www.ncbi.nlm.nih.gov/variation/tools/1000genomes/); GO-ESP, NHLBI GO Exome Sequencing Project (http://evs.gs.washington.edu/EVS/); GnomAD, genome Aggregation Database (http://gnomad.broadinstitute.org/)
